# The War Fund

**Published:** 1899-12-02

**Authors:** Valentine Matthews

**Affiliations:** Surgeon-Major V.M.S.C., Hon. Secretary V.M.S.C. Relief Fund. Headquarters, London Companies V.M.S.C., 51, Calthorpe Street, W.C.


### Editor's Letter-Box.

[Our Correspondents are reminded that prolixity is a great bar to publication, and that brevity of style and conciseness of statement greatly facilitate early insertion.]

### THE WAR FUND.

We have received the following letter: Sir,—Will you allow me through the medium of your columns to appeal to the medical profession for subscriptions to a fund now being raised by the London Companies' Volunteer Medical Staff Corps for the benefit of the wives and families of non-commissioned officers and men of the Royal Army Medical Corps on active service in South Africa? The fund is being raised with the approval of the Director-General of the Army Medical Department, and with the active support of Surgeon Lieut.-Colonel J. E. Squire, commanding the London companies. A committee, of which he is the chairman, has been formed among the officers of the corps. All monies collected will be handed over to the Depôt Mobilisation Relief Fund of the Royal Army Medical Corps at Aldershot, which is working in connection with the Soldiers and Sailors' Families Association, and relieves the wives and families either "on or off the strength" of any case belonging to the R.A.M. Corps, whether the husbands were mobilised at Aldershot or not. It is needless to remind members of the profession that the men of the R.A.M. Corps, although non-combatants, are frequently exposed to fire, and share all the fatigues and hardships of combatant corps, besides the additional risk of infection incurred in nursing the sick, and that by subscribing to this fund they will show in a practical way their appreciation of the work done by this branch of the service, and their sympathy with the men's families. In addition to volunteer medical officers present and past, former members of the V.M.S.C., and retired medical officers of the services, may I appeal especially to civilian practitioners and all interested in civilian medical institutions to help the homes of those men who are "doing the country's work" in a department which has the first claim on the medical profession? Subscriptions sent to me, addressed as below, will be gratefully acknowledged.—I am, Sir, yours faithfully,

Valentine Matthews, Surgeon-Major V.M.S.C.,
Hon. Secretary V.M.S.C. Relief Fund.

Headquarters, London Companies V.M.S.C.,
51, Calthorpe Street, W.C., Nov. 25th, 1899.

